# Improving birth outcomes for women who are substance using or have mental illness: a Canadian cohort study comparing antenatal midwifery and physician models of care for women of low socioeconomic position

**DOI:** 10.1186/s12884-019-2428-y

**Published:** 2019-08-06

**Authors:** Daphne N. McRae, Nazeem Muhajarine, Patricia A. Janssen

**Affiliations:** 1 Department of Community Health and Epidemiology, University of Saskatchewan, Box 7 Health Science Building 107 Wiggins Road, Saskatoon SK, S7N 5E5 Canada; 2 School of Population and Public Health, University of British Columbia, 2206 East Mall, Vancouver BC, V6T 1Z3 Canada

**Keywords:** Midwifery, Mental health, Substance use, Preterm birth, Small-for-gestational-age, Socioeconomic position, Health services research

## Abstract

**Background:**

Some observational studies have shown improved birth outcomes for women of low socioeconomic position (SEP) receiving antenatal midwifery versus physician care. To understand for whom and under what circumstances midwifery care is associated with better birth outcomes we examined whether psychosocial risk including substance use, mental illness, social assistance, residence in a neighbourhood of low/moderate SEP, and teen maternal age modified the association between model of care (midwifery versus physician) and small-for-gestational-age (SGA) or preterm birth (PTB) for women of low SEP.

**Methods:**

For this retrospective cohort study, maternity data from the British Columbia Perinatal Data Registry were linked with Medical Services Plan billing data. We report adjusted odds ratios (aORs) and 95% confidence intervals (CIs) for SGA birth (< the 10th percentile) and PTB (< 37 weeks’ completed gestation). For tests of interaction between antenatal models of care and psychosocial risk, *p*-values < 0.10 were considered statistically significant. Women were eligible for inclusion if they were residents of British Columbia, Canada, carried a singleton fetus, had low to moderate medical/obstetric risk, birthed between April 1, 2008 and Dec. 31, 2012, and received a health insurance subsidy (*n* = 33,937).

**Results:**

Midwifery versus obstetrician patients had lower odds of PTB. The difference was 31% larger among substance users (aOR 0.24, 95% CI: 0.11–0.54) compared to non-substance users (aOR 0.55, 95% CI: 0.45–0.68). Additionally, there was a 34% statistically significant absolute difference in odds of PTB for midwifery versus obstetrician patients with both mental illness and substance use (aOR 0.18, 95% CI: 0.06–0.55) compared to women with neither mental illness nor substance use (aOR 0.52, 95% CI: 0.41–.66). Results demonstrated a consistent association between midwifery versus physician care and lower odds of SGA, yet effects were not statistically significantly different for women with higher or lower psychosocial risk.

**Conclusion:**

Among low SEP women in British Columbia, Canada, antenatal midwifery compared to obstetrician care was associated with reduced odds of PTB. Odds were lower among women with substance use, and mental illness and substance use, than among women without these risk factors.

**Electronic supplementary material:**

The online version of this article (10.1186/s12884-019-2428-y) contains supplementary material, which is available to authorized users.

## Introduction

A Cochrane Review published in 2016, involving eight trials (*n* = 13,238) in high income countries, demonstrated a 24% reduction in preterm birth (risk ratio: 0.76, 95% confidence interval (CI): 0.64, 0.91) for women randomized to receive perinatal care from a single midwife or a small call group compared to women in other models of care (e.g. physician-led, or midwifery-physician models) [1]. After examining the review, the World Health Organization reported that the evidence in support of this finding is of moderate certainty [2]. In response, the WHO has recommended access to “well-functioning midwifery programs” ([2], p89) as a health systems’ intervention for improving utilization and quality of antenatal care.

Some observational studies specifically focusing on women of low socioeconomic position (SEP) have also demonstrated better birth outcomes for midwives’ versus physicians’ patients [3]. We recently published a retrospective cohort study from British Columbia (BC), Canada, demonstrating a 29 to 41% reduction in odds of small-for-gestational-age (SGA) birth and a 26 to 47% reduction in odds of preterm birth (PTB) for midwifery versus general practitioner (GP) or obstetrician (OB) patients of low SEP who had low to moderate perinatal risk [4]. The aim of this current analysis is to understand for whom and under what circumstances midwifery care is associated with better birth outcomes. Therefore, we examined whether psychosocial risk including substance use, mental illness, social assistance, residence in a neighbourhood of low/moderate socioeconomic position, and teen maternal age modified the association between model of care (midwifery versus physician) and small-for-gestational-age (SGA) or preterm birth (PTB) for women of low SEP.

## Methods

This section provides a summary of the previously published, full study protocol [4].

### Setting

In BC, women self-select their maternity provider (generally GPs, OBs, or midwives) subject to availability. Women with no perinatal complications or low to moderate pregnancy risk (as defined by the BC College of Midwives’ guidelines [5]) are eligible to receive care from any type of registered provider, with fees paid by the provincial Medical Services Plan (MSP). Midwives are required to consult with a physician (usually an OB) for moderate pregnancy complications and to transfer care to an OB for high-risk complications [5]. Midwifery care in Canada is based on a relational model where care is provided by a single midwife or small pool of midwives known to a client and accessible by phone 24 h a day [6]. The midwifery model emphasizes holistic care, continuity of care provider, and informed choice (particularly concerning medical interventions and birth location). Midwives have a capped annual caseload and are paid per full or partial trimester of care [6] enabling lengthy antenatal visits (30 to 60 min on average [7]).

### Study design

For this retrospective, population-level cohort study we assessed the association between antenatal models of care and SGA and PTB for low SEP women with and without specific psychosocial risk factors. Model of care was determined by linking women’s maternity records to practitioners’ MSP billing records (billing records indicate the type(s) of practitioner(s) involved in antenatal care). Maternity data, including data on mental health, substance use, and teen maternal age, were obtained from the BC Perinatal Data Registry (PDR) [8], which includes data from hospital and home birth records as well as International Statistical Classification of Diseases, Tenth Revision, Canada (ICD-10-CA) codes from the Canadian Institutes of Health Information Discharge Abstract Database. Approximately 99% of all BC births are recorded in the PDR [9] with a chart re-abstraction study showing high validity on key surveillance variables [10].

PDR data were linked to MSP billing data [11] to determine if women were of low SEP and to identify women receiving social assistance (public financial aid for low income). Low SEP was operationalized as receipt of a regular MSP health insurance subsidy during the year of delivery [11]. Subsidy assistance is offered to low income individuals based on their previous year’s household income (e.g. a family of three earning $28,000 or less in 2008/2009 or $30,000 or less in 2010–2012 would have been eligible for a health insurance subsidy) [11]. Women receiving social assistance have their health insurance premiums waived and this information is also recorded in the MSP billing data.

Maternity and billing data were linked with neighbourhood demographic data provided by Population Data BC [12], and Local Health Area (LHA) socioeconomic and income inequality rankings publicly available from BC’s Division of Statistics [13, 14]. Variables from these sources were tested as modifying (neighbourhood SEP) or confounding factors (LHA socioeconomic rank and LHA income inequality).

### Study sample

Our study included pregnant, low SEP women who were residents of BC, received antenatal midwifery, GP, or OB care, carried a singleton fetus, were eligible for midwifery care throughout the antenatal period (having no or low to moderate medical/obstetric risk according to midwifery guidelines [5]), birthed between April 1, 2008 and Dec. 31, 2012, received an MSP health insurance subsidy, and were not registered Status Indian^1^ (*n* = 33,937). (‘Status Indian’ is a legal term referring to the Indigenous identity of people registered under the Canadian government’s *Indian Act* and eligible for certain government benefits and services [15]). Status Indian women were excluded from the study because they had their health insurance premiums paid by Health Canada and therefore were not eligible for an MSP health insurance subsidy.

Moderate medical and/or obstetric risk (defined in Table 1) included conditions which would require a midwife to consult with an OB yet allow a midwife to retain the primary care provider role. As OB patients can have higher pregnancy risk than midwifery clients we excluded all physicians’ patients who had antenatal conditions rendering them ineligible for midwifery care. Likewise, we excluded all midwifery clients who required a transfer to OB care during the antenatal period. Only eligible cases with complete data were included in the analyses (99.79% of the cases for SGA and 99.87% for PTB).

**Table 1:** Frequencies and proportions of maternal characteristics by antenatal model of care, British Columbia, April 1, 2008-December 31, 2012 (*n* = 33,937)

Maternal Characteristics	Antenatal Model of Care		
	MW *n* = 3397 *n*(%)	GP *n* = 25,784 *n*(%)	OB *n* = 4756 *n*(%)
Age (yrs.)			
14–19	117 (3.44)	2587 (10.03)	208 (4.37)
20–24	644 (18.96)	8366 (32.45)	841 (17.68)
25–29	1161 (34.18)	7716 (29.93)	1371 (28.83)
30–34	987 (29.06)	4559 (17.68)	1247 (26.22)
35–39	415 (12.22)	2113 (8.20)	808 (16.99)
> 40	73 (2.15)	443 (1.72)	281 (5.91)
Parity^a^			
Nullipara	1607 (47.31)	13,148 (50.99)	2134 (44.87)
Multipara	1790 (52.69)	12,636 (49.01)	2621 (55.11)
Medical risk^b,c^	11 (0.32)	263 (1.02)	90 (1.89)
Prior obstetric risk^b,d^	87 (2.56)	999 (3.87)	280 (5.89)
Mental illness^b,e^	1012 (29.79)	4922 (19.09)	545 (11.46)
Receiving social assistance^b^	239 (7.04)	3623 (14.05)	535 (11.25)
Pre-pregnancy Body Mass Index^f^			
Underweight	156 (4.59)	1281 (4.97)	277 (5.82)
Normal	1854 (54.58)	9616 (37.29)	1652 (34.74)
Overweight	511 (15.04)	3554 (13.78)	528 (11.10)
Obese	243 (7.15)	2340 (9.08)	327 (6.88)
Unknown	633 (18.63)	8993 (34.88)	1972 (41.46)
Smoking status			
Never	915 (26.94)	5189 (20.12)	1325 (27.86)
Former	548 (16.13)	3456 (13.40)	299 (6.29)
Current	336 (9.89)	5540 (21.49)	459 (9.65)
Unknown	1598 (47.04)	11,599 (44.99)	2673 (56.20)
Substance use in pregnancy^b,g^	153 (4.50)	2281 (8.85)	182 (3.83)
Alcohol identified as a risk^b^	47 (1.38)	753 (2.92)	31 (0.65)
Antepartum morbidity^b,h^	243 (7.15)	3863 (14.98)	1189 (25.00)
Delivery year			
2008 (as of April 1)	419 (12.33)	4495 (17.43)	711 (14.95)
2009	606 (17.84)	5640 (21.87)	910 (19.13)
2010	694 (20.43)	5371 (20.83)	1000 (21.03)
2011	796 (23.43)	5337 (20.70)	1014 (21.32)
2012	882 (25.96)	4941 (19.16)	1121 (23.57)
Neighbourhood SEP^i^			
High	450 (13.25)	2846 (11.04)	377 (7.93)
Low/Medium	2947 (86.75)	22,938 (88.96)	4379 (92.07)
LHA Socioeconomic Rank^j^			
High (Best)	1858 (54.70)	7314 (28.37)	2327 (48.93)
Medium	1118 (32.91)	12,774 (49.54)	1897 (39.89)
Low	412 (12.13)	5641 (21.88)	487 (10.24)
Unknown	9 (0.26)	55 (0.21)	45 (0.95)
LHA Income Inequality Rank^k^			
High (Worst)	1188 (34.97)	5776 (22.40)	2419 (50.86)
Medium	1717 (50.54)	14,829 (57.51)	1999 (42.03)
Low	483 (14.22)	5133 (19.91)	318 (6.69)
Unknown	9 (0.26)	46 (0.18)	20 (0.42)
Northern residence^b,l^	92 (2.71)	3426 (13.29)	205 (4.31)

Abbreviations: *GP* general practitioner, *MW* midwife, *OB* obstetrician, *SEP* socioeconomic position

^a^Missing cases amount to 5 or less

^b^Values represent cases classified as “Yes”, the remainder of the cases were classified as “No”, “Unknown”, or were undocumented

^c^Included maternal disease of the respiratory or digestive system, and endocrine, nutritional, or metabolic disease

^d^Included women with at least one of the following conditions in past pregnancy: infant with major congenital anomaly, neonatal death, stillbirth, or one preterm delivery

^e^Included any of the following diagnoses prior to, or during the current pregnancy: anxiety disorder, depression, postpartum depression, bipolar disorder, other/unknown (including schizophrenic, mood, and psychotic disorders)

^f^Classified according to Health Canada’s guidelines [34]

^g^Heroin/opioids, cocaine, methadone, solvents, marijuana, or other/unknown drugs used at any time during pregnancy, prescription or other drug use identified as a risk at any time during pregnancy

^h^Included pregnancy induced hypertension, gestational diabetes (whether or not insulin dependent), anemia, intrauterine growth restriction, viral disease, infection and parasitic disease, placenta previa without hemorrhage, polyhydramnios or oligohydramnios, antepartum hemorrhage > 20 weeks, sexually transmitted infection or HIV, or premature separation of the placenta

^i^Neighbourhood income quintiles were classified as low/medium (quintiles 1–4) versus high (quintile 5) [12]

^j^Calculated by the province of BC’s Statistics Division (BC Stats), based on a range of social determinants of health reflecting area-level economic and social processes, and policy decisions [14]

^k^Calculated by BC Stats [14]

^l^At the time of delivery, normal residence in BC’s Northern Health Authority

### Outcomes

Our primary outcome was SGA birth (< 10th percentile) according to Kierans’ et al. sex-specific birth weight references. The reference tables are applicable for singleton, live-born infants between 20 and 44 weeks’ completed gestation. They specify threshold values for infant weight by gestational age which equate to less than the 10th percentile of provincial birth weight distributions. This birth weight reference is the most appropriate tool for classifying SGA birth in our study as it was constructed using population-based birth weight distributions from BC between 1981 and 2000, and was approved for use in BC hospitals as of 2004 [16]. PTB was defined as birth less than 37 weeks’ completed gestation.

### Statistical analyses

Data was analyzed using multivariable, logistic regression models and a Generalized Estimating Equation (GEE) approach to account for clustering of effects by siblings and by community [17]. Women self-selected their model of care therefore those who knew they had higher pregnancy risk (within the low to moderate risk spectrum) could have chosen OB care more often resulting in selection bias. To address this concern we conducted two sensitivity analyses. First, we controlled for select antepartum morbidities (see definition in Table 1) which could be associated with medical conditions, such as uterine anomaly, undocumented in the PDR. Second, we excluded all patients with prior medical or obstetric risk to assess if differences in severity of known risk factors between practitioner-types could explain the observed associations.

To investigate if psychosocial risk (substance use, mental illness, social assistance, low/moderate neighbourhood SEP, teen maternal age) modified the association between model of care and SGA or PTB, interaction terms comprised of model of care multiplied by each type of risk were included in the GEE models. The effect of *combined* psychosocial risk was tested using two-way interactions, e.g. “model of care x mental illness and substance use” (defined as women with both versus neither). We report adjusted odds ratios (aORs) and 95% CIs for the association between model of care and SGA/PTB. For tests of interaction we report *p*-values. For interactions we considered *p* < 0.10 as statistically significant because the aim was to identify clinically important differences between subgroups, if any, within the larger sample and therefore maximize possibilities for tailored interventions [18]. Our assessments for effect modification were based on the statistical significance of the interactions, the consistency between the direction of the main effect estimates and subgroup effects, patterns of effect for different types of psychosocial risk, and significance of the subgroup aORs [19]. SAS Enterprise 7.1 was utilized for all data analyses [20].

## Results

There were 3397 midwifery, 25,784 GP and 4756 OB pregnancies included in the analyses (Table 1). Midwifery clients had significantly higher prevalence of mental illness (29.79%) compared to GP (19.09%) or OB patients (11.46%). Depression was the most frequent mental health condition reported. The prevalence of depression, as indicated in the maternal record, was much greater among midwives’ clients (18.55%) than GPs’ (12.60%) or OBs’ patients (7.44%). Substance use was more frequently reported among GPs’ patients (8.85%) than either midwives’ (4.50%) or OBs’ patients (3.83%). Likewise, there was a greater proportion of teen mothers attended during the antenatal period by GPs (10.03%) than by OBs (4.37%) or midwives (3.44%). GPs also cared for a greater proportion of women receiving social assistance (14.05%) than either OBs (11.25%) or midwives (7.04%). Most women lived in low or middle SEP neighbourhoods, with a slightly larger proportion of midwifery clients living in high SEP neighbourhoods (13.25% versus 11.04% for GPs’ and 7.93% for OBs’ patients).

### Small-for-gestational-age birth

Of the 33,866 eligible births included in the analysis, 2378 (7.02%) were SGA (Table 2). On average, women of low SEP receiving antenatal midwifery care, compared to GP care, had lower adjusted odds of SGA birth (aOR 0.73, 95% CI: 0.62–0.86). (Model adjusted for maternal age, parity, pre-pregnancy BMI, infant sex, smoking status, substance use, mental illness/disorder, and Local Health Area socioeconomic rank). Likewise, midwifery versus OB patients were less likely to have an SGA birth (aOR 0.60, 95% CI: 0.49–0.72) as were GP versus OB patients (aOR 0.82: 95% CI: 0.73–0.93). When adjusting for select antepartum morbidities (see definition in Table 1) and when excluding patients with prior obstetric or medical risk to control for the possibility of OB patients having systematically higher pregnancy risk on the low to moderate risk spectrum, midwifery clients continued to have significantly more favourable birth outcomes than physicians’ patients (see results of the sensitivity analyses in Additional file 1).

**Table 2:** Frequencies and adjusted odds ratios for small-for-gestational-age birth by antenatal model of care and psychosocial risk characteristics, British Columbia, April 1, 2008-December 31, 2012

Small-for-gestational-age birth by model of care						
	MW *n* = 3391	GP n = 25,733	OB *n* = 4742	MW vs. GP	MW vs. OB	GP vs. OB
	*n*(%)	*n*(%)	*n*(%)	aOR (95% CI)	aOR (95% CI)	aOR (95% CI)
Mental illness^a^						
Yes	42/1011 (4.15)	340/4912 (6.92)	45/545 (8.26)	0.64 (0.46–0.89)	0.49 (0.32–0.77)	0.77 (0.55–1.08)
No	129/2380 (5.42)	1461/20,821 (7.02)	361/4197 (8.60)	0.76 (0.63–0.92)	0.63 (0.51–0.78)	0.83 (0.73–0.94)
Substance use^b^						
Yes	6/152 (3.95)	222/2275 (9.76)	23/182 (12.64)	0.41 (0.18–0.93)	0.31 (0.12–0.78)	0.75 (0.46–1.21)
No	165/3239 (5.09)	1579/23,458 (6.73)	383/4560 (8.40)	0.75 (0.63–0.89)	0.62 (0.51–0.75)	0.83 (0.73–0.94)
Teen mother						
Yes (14–19 yrs.)	8/117 (6.84)	181/2582 (7.01)	21/205 (10.24)	0.94 (0.45–1.98)	0.56 (0.24–1.34)	0.60 (0.36–0.99)
No (25–29 yrs.)	55/1159 (4.75)	537/7697 (6.98)	126/1370 (9.20)	0.71 (0.53–0.94)	0.55 (0.39–0.76)	0.78 (0.63–0.96)
Mental illness, substance use						
Both	5 or less /95^c^	90/927 (9.71)	10/76 (13.16)	0.33 (0.10–1.07)	0.22 (0.06–0.87)	0.68 (0.32–1.43)
Neither	126/2322 (5.43)	1324/19,424 (6.82)	348/4086 (8.52)	0.78 (0.64–0.94)	0.64 (0.51–0.79)	0.82 (0.72–0.94)

Abbreviations: *MW* midwife, *GP* general practitioner, *OB* obstetrician, *OR* odds ratio, *CI* confidence interval

^a^Mental illness included any of the following diagnoses prior to, or during the current pregnancy: anxiety disorder, depression, postpartum depression, bipolar disorder, other/unknown (including schizophrenic, mood, and psychotic disorders). Aside from mild anxiety or depression, a physician would diagnose mental illness

^b^Substance use included any indication in the medical record of heroin/opioids, cocaine, methadone, solvents, marijuana or other/unknown drugs used by the mother at any time during pregnancy, as well as prescription or other drug use identified as a risk by the provider

^c^Percentage suppressed due to small cell size

*Outcomes had statistically significant tests of interaction (*p* < 0.10) comparing difference of effect across strata (yes vs. no)

Models adjusted for all variables listed except stratifying variables: maternal age, parity, pre-pregnancy BMI, infant sex, smoking status, substance use, mental illness/disorder, and Local Health Area (LHA) socioeconomic rank

Odds ratios based on 2378 births with SGA and 33,866 total births with no missing information for this analysis

Adjusted odds of SGA for midwifery versus GP or OB patients were lower for those with mental illness, substance use, or mental illness and substance use, compared to women without these psychosocial risk factors. Yet the difference in effect estimates were not statistically significant for those with higher versus lower psychosocial risk. For example, both substance users (aOR 0.41, 95% CI: 0.18–0.93) and non-substance users (aOR 0.75, 95% CI: 0.63–0.89), had significantly *lower odds of SGA* if in the care of midwives versus GPs (Table 2), however odds ratios were *not significantly different* (*p* = 0.16) across the substance user and non-user groups.

Likewise for midwifery versus OB patients, odds of SGA were 31% lower among substance users compared to non-substance users (aOR 0.31, 95% CI: 0.12–0.78 versus aOR 0.62, 95% CI: 0.51–0.75), but tests of interaction did not indicate a significant difference in odds by substance user versus non-user strata (*p* = 0.15). When testing the modifying effect of mental illness (presence versus absence), teen maternal age (14–19 versus 25–29), and mental illness and substance use (both versus neither) on SGA by model of care there was no evidence of effect modification.

### Preterm birth

Overall, preterm birth occurred in 6.43% (*n* = 2178) of the eligible study sample (*n* = 33,893) (Table 3). The adjusted odds of PTB was statistically significantly smaller for woman of low SEP receiving antenatal care from midwives versus GPs (aOR 0.79, 95% CI: 0.66–0.94) and midwives versus OBs (aOR 0.53, 95% CI: 0.43–0.64). Models were adjusted for maternal age, medical risk, prior obstetric risk, pre-pregnancy BMI, infant sex, receipt of social assistance, smoking status, substance use, mental illness/disorder, neighbourhood SEP, Local Health Area socioeconomic rank, and northern residence. On average, GP patients were also less likely to have a PTB than OB patients (aOR 0.67, 95% CI: 0.59–0.75).

**Table 3:** Frequencies and adjusted odds ratios for preterm birth by antenatal model of care and psychosocial risk characteristics, British Columbia, April 1, 2008-December 31, 2012

Preterm birth by model of care						
	MW *n* = 3394	GP *n* = 25,753	OB *n* = 4746	MW vs. GP	MW vs. OB	GP vs. OB
	*n*(%)	*n*(%)	*n*(%)	aOR (95% CI)	aOR (95% CI)	aOR (95% CI)
Mental illness^a^						
Yes	56/1011 (5.54)	344/4917 (7.00)	71/545 (13.03)	0.92 (0.68–1.24)	0.49 (0.34–0.72)	*0.54 (0.41–0.71)
No	98/2383 (4.11)	1257/20,836 (6.03)	352/4201 (8.38)	0.73 (0.59–0.91)	0.51 (0.41–0.65)	0.70 (0.61–0.79)
Substance use^b^						
Yes	8/152 (5.26)	215/2276 (9.45)	39/182 (21.43)	0.59 (0.29–1.21)	*0.24 (0.11–0.54)	*0.41 (0.28–0.61)
No	146/3242 (4.50)	1386/23,477 (5.90)	384/4564 (8.41)	0.80 (0.67–0.96)	0.55 (0.45–0.68)	0.69 (0.61–0.79)
Teen mother						
Yes (14–19 yrs.)	9/117 (7.69)	187/2584 (7.24)	21/206 (10.19)	1.09 (0.55–2.19)	0.74 (0.33–1.66)	0.67 (0.42–1.09)
No (25–29 yrs.)	46/1161 (3.96)	475/7705 (6.16)	100/1371 (7.29)	0.69 (0.50–0.94)	0.56 (0.39–0.80)	0.81 (0.65–1.02)
Social assistance^c^						
Yes	17/239 (7.11)	299/3617 (8.27)	61/534 (11.42)	0.89 (0.53–1.50)	0.63 (0.36–1.12)	0.71 (0.53–0.96)
No	137/3155 (4.34)	1302/22,136 (5.88)	362/4212 (8.59)	0.78 (0.64–0.94)	0.51 (0.42–0.63)	0.66 (0.58–0.75)
Neigh. SEP^d^						
Low/Medium	135/2944 (4.59)	1429/22,915 (6.24)	397/4369 (9.09)	0.80 (0.66–0.96)	0.52 (0.42–0.64)	0.65 (0.58–0.74)
High	19/450 (4.22)	172/2838 (6.06)	26/377 (6.90)	0.72 (0.44–1.17)	0.63 (0.34–1.17)	0.88 (0.57–1.36)
Mental illness, substance use						
Both	5 or less /95^e^	95/927 (10.25)	18/76 (23.68)	0.43 (0.15–1.19)	*0.18 (0.06–0.55)	*0.41 (0.23–0.74)
Neither	94/2325 (4.04)	1134/19,438 (5.83)	331/4090 (8.09)	0.73 (0.59–0.91)	0.52 (0.41–0.66)	0.71 (0.62–0.81)
Mental illness, social assistance						
Both	7/128 (5.47)	117/1195 (9.79)	27/161 (16.77)	0.58 (0.26–1.32)	0.35 (0.14–0.86)	0.60 (0.38–0.96)
Neither	88/2272 (3.87)	1075/18,414 (5.84)	318/3828 (8.31)	0.70 (0.56–0.88)	0.48 (0.37–0.61)	0.68 (0.59–0.79)
Substance use, social assistance						
Both	5 or less /45^e^	94/832 (11.30)	22/93 (23.66)	0.78 (0.27–2.27)	0.34 (0.11–1.10)	0.44 (0.26–0.76)
Neither	133/3047 (4.36)	1178/20,643 (5.71)	345/4118 (8.38)	0.78 (0.65–0.95)	0.53 (0.43–0.65)	0.67 (0.59–0.77)

Abbreviations: *MW* midwife, *GP* general practitioner, *OB* obstetrician, *OR* odds ratio, *CI* confidence interval

^a^Mental illness included any of the following diagnoses prior to, or during the current pregnancy: anxiety disorder, depression, postpartum depression, bipolar disorder, other/unknown (including schizophrenic, mood, and psychotic disorders). Aside from mild anxiety or depression, a physician would diagnose mental illness

^b^Substance use included any indication in the medical record of heroin/opioids, cocaine, methadone, solvents, marijuana or other/unknown drugs used by the mother at any time during pregnancy, as well as prescription or other drug use identified as a risk by the provider

^**c**^Social assistance recipients received public financial assistance during the year of delivery due to low income

^d^Neighbourhood low/medium socioeconomic position included women residing in the four lowest income quintiles, depending on residential postal code at delivery

^e^Percentage suppressed due to small cell size

*Outcomes had statistically significant tests of interaction (*p* < 0.10) comparing difference of effect across strata (yes vs. no)

Models adjusted for all variables listed except stratifying variables: maternal age, medical risk, obstetric risk, pre-pregnancy BMI, infant sex, receipt of social assistance, smoking status, substance use, mental illness/disorder, neighbourhood SEP, Local Health Area socioeconomic rank, and northern residence

Odds ratios based on 2178 PTB births and 33,893 total births with no missing information for this analysis

**Table 4:** Post-hoc power estimates for moderation analyses investigating antenatal models of care by small-for-gestational-age birth and pre-term birth (alpha set at 0.10)

Moderating Factor	MW vs. GP (%)	MW vs. OB (%)	GP vs. OB (%)
Small-for-Gestational-Age Birth			
Mental illness	93.96	94.63	28.17
Substance use	72.05	83.46	29.69
Teen mother	6.43	19.80	47.04
Mental illness, substance use^a^	–	–	18.25
Preterm Birth			
Mental illness	48.79	99.97	99.95
Substance use	47.40	99.37	99.96
Teen mother	6.96	12.70	40.57
Social assistance	12.50	52.39	75.36
Neigh. SEP	96.77	100	100
Mental illness, substance use^a^	–	–	95.83
Mental illness, social assistance	41.30	87.37	82.03
Substance use, social assistance^a^	–	–	94.62

Abbreviations: *MW* midwife, *GP* general practitioner, *OB* obstetrician, *Neigh* neighbourhood

^a^Percentage suppressed due to low frequency of cases

When assessing residual confounding by excluding women with prior obstetric risk or medical risk from the analysis, the odds of PTB remained significantly lower for midwives’ versus physicians’ patients (see results in Additional file 1). This provides evidence of an effect by model of care, independent of perinatal risk. When controlling for morbidity arising during the antenatal period, results demonstrated statistically significant reductions in odds of PTB for midwives’ versus OBs’ patients and GPs’ versus OBs’ patients but no significant difference in odds for midwives’ versus GPs’ patients. Slightly higher prevalence of antenatal morbidity for GPs’ versus midwives’ patients may help to explain the lower odds of PTB midwives’ clients experienced. However when we conducted the same sensitivity analysis with a larger sample of low SEP women (*n* = 57,763) for our main study, results showed significantly lower odds of PTB for midwives’ versus GPs’ patients [4], suggesting a lack of power to detect differences in effect for PTB within this smaller sample.

Substance using women in the care of midwives versus OBs had 31% significantly lower odds of PTB compared to non-substance users (aOR 0.24, 95% CI: 0.11–0.54 versus aOR 0.55, 95% CI: 0.45–0.68) (Table 3). Tests showed evidence of effect modification (*p* = 0.05).

For GP versus OB patients, substance use also significantly modified the relationship between antenatal model of care and PTB. GP versus OB patients had 28% significantly lower odds of PTB compared to non-substance users (aOR 0.41, 95% CI: 0.28–0.61 versus aOR 0.69, 95% CI: 0.61–0.79). GP versus OB care was associated with 16% significantly lower odds of PTB among women with mental illness compared to women without mental illness (aOR 0.54, 95% CI: 0.41–0.71 versus aOR 0.70, 95% CI: 0.61–0.79), indicating effect modification.

Midwifery versus OB patients with both mental illness and substance use had 34% significantly lower odds of PTB (aOR 0.18, 95% CI: 0.06–0.55) compared to those with neither risk factor (aOR 0.52, 95% CI: 0.41–0.66). Comparing GP versus OB patients, odds of PTB were 30% significantly lower for those with mental illness and substance use compared to those without these risks (aOR 0.41, 95% CI: 0.23–0.74 versus aOR 0.71, 95% CI: 0.62–0.81). None of the psychosocial risk characteristics examined modified the relationship between midwifery versus GP care and PTB (Table 3).

### Post-hoc power estimates

Despite having all population-level data available for eligible midwifery clients in BC during the study period, the sample sizes used to compare modifying factors were small, particularly for the midwifery cohort, potentially impacting the power to detect differences between groups for some outcomes. Power estimations for this study were not conducted prior to the analyses as there have been no published estimates of adverse outcomes among women of low SEP receiving midwifery care in Canada. Post-hoc power estimates indicate low power (< 80%, alpha set at 0.10) for a number of comparisons (Table 4). However, the SGA and PTB analyses were adequately powered to detect differences between midwifery and obstetrician care, modified by mental illness or substance use. Likewise, there was adequate power to assess the association between midwifery versus GP care and SGA, modified by mental illness.

## Discussion

In this study, including only women of low socioeconomic position, the odds of SGA and PTB were lower among those receiving antenatal care from midwives versus OBs and the association between midwifery care and PTB was stronger among women using substances compared to non-users. Midwifery versus OB patients with both mental illness and substance use also had lower odds of PTB than those with neither mental illness nor substance use. Comparing antenatal GP versus OB care, there were lower odds of PTB among GP patients with mental illness, substance use, and combined mental illness and substance use compared to women without these vulnerabilities. The reduction in odds of PTB within subgroups of substance users and substance users with mental health conditions was greatest for midwifery versus OB patients, followed by GP versus OB patients. Exposure to the psychosocial risk characteristics examined did not modify the associations between midwifery care and SGA or PTB, compared to GP care.

Previous midwifery/physician studies conducted in high resource countries examining antenatal model of care and poor birth outcomes for women of low SEP have examined only the modifying effect of age [3]. A randomized controlled trial from the USA examined the relationship between low birth weight (LBW) for low SEP women receiving enhanced antenatal care from nurse-midwives versus standard care from OB residents, stratified by age (< 16, 17–19, > 20 years) [21]. Similar to our SGA findings, their results did not indicate a difference in effect in LBW for nurse-midwifery versus physician care by teen maternal age. Likewise, a retrospective cohort study which investigated LBW and very LBW among American Medicaid recipients receiving antenatal nurse-midwifery versus physician care found no difference in relative risk for teen mothers compared to women under 40 [22].

This study was limited to the analysis of variables available in the administrative database. We did not have access to information on mother’s race/ethnicity (including Status Indian), education, and measures of maternal health knowledge and attitudes/values. These factors have shown to be important characteristics associated with antenatal model of care and independent risk factors for adverse birth outcomes. However, it should be noted that our sample was restricted to prenatal women with low income (thereby controlling for effects due to SEP), and we were able to assess and/or control for other factors including smoking and alcohol use during pregnancy, and pre-pregnancy BMI. Our findings were unlikely to be biased by our inability to assess outcomes among women who were Status Indian as they represented approximately 2% of the female population in BC during the study period [23].

Despite the WHO endorsing midwifery care as a possible means of reducing the risk of PTB, they acknowledge that the mechanisms linking midwifery care to improved outcomes are currently “unclear” ([2], p90). Likewise, for women of low SEP the pathways between antenatal midwifery care and diminished odds of SGA and PTB are undetermined. However, factors that may positively mitigate the association include improved self-care (e.g. nutritional habits), increased prenatal care utilization and follow-through on clinical advice, and greater self-efficacy—due to the additional time given at the care encounter, emotional support, and depth of relationship the midwifery model affords. Women of low SEP using substances, or having mental illness and substance use, may struggle with low self-esteem and feelings of worthlessness reflecting their stigmatized social position [24, 25]. This could affect birth outcomes by impeding motivation for self-care and impacting nutritional habits, prenatal care utilization, and follow-through on clinical advice [26, 27]. The explicit emphasis on dignity and empowerment within the midwifery model [28] may help to dispel unhealthy self-concepts and expectations of clinician judgement, frequently reported by individuals of low SEP [29], thus lowering the prevalence of higher risk behaviour (e.g. self-medicating). The midwifery model encourages women to maintain “agency” described as the autonomy and empowerment that exist when women retain control as the primary-decision makers in their own care ([30], p26). Promoting informed choice, midwives work “in partnership with women to strengthen women’s own capabilities to care for themselves and their families” ([31], p3). By facilitating ownership over pregnancy, health, and lifestyle choices, the midwifery model may foster self-efficacy—the belief that one has the ability to effect personal change or reach goals [32, 33] —a pre-cursor to cessation or reduction of high risk behaviour (e.g. substance use).

Interestingly, there was higher prevalence of mental health conditions recorded in the maternity record among midwives’ clients (29.79%) compared to physicians’ patients (GP 19.09%; OB 11.46%). This may be because midwifery clients were more willing to disclose mental health concerns due to greater clinician-client trust cultivated through continuity of care and longer appointments. Or, midwives may have practiced more holistic care (e.g. inquiring about emotional well-being throughout pregnancy) and have had greater opportunity for clinical observation. If mental health diagnosis was greater among midwifery clients due to the antenatal care model, then midwifery care may have been more conducive to the prevention of infant morbidity through mental health treatment, explaining in part the lower odds of PTB for midwifery versus obstetrician patients with psychosocial risk.

## Conclusion

Among low-income prenatal women, those experiencing psychosocial risk including substance use, or mental illness and substance use, have lower rates of PTB if receiving midwifery versus OB care. A strength of this study is that it explicitly highlights differences in birth outcomes among women with low SEP and other psychosocial risk attributes, by model of care. However, more research is needed to determine how differing antenatal models of care impact women’s and newborn’s health. Future studies should examine aspects of antenatal maternity models that diverge (e.g. length of appointments, emotional support, mental health screening, behavioural/nutritional counselling, self-care and empowerment education) and how these factors contribute to variation in outcomes. In addition, further insight may be gleaned from a comparison of characteristics of midwifery and GP models of care that coincide but differ from OB care as results indicated similarity in outcomes among midwives and GPs in contrast to OBs. Our study should be replicated with larger midwifery samples to further explore modification of effects within strata of psychosocial risk. This could further provide guidance on strategies for enhancing and tailoring prenatal care according to women’s specific needs.

## Additional file

Additional file 1: Sensitivity Analyses. This file (appendix_a_sensitivity_analyses.pdf) contains results of two sensitivity analyses, displayed in Table 1: Adjusted odds ratios and 95% CIs without and with control for antepartum morbidity and Table 2: Adjusted odds ratios and 95% CIs for full study population and for study population excluding pregnancies in which mothers had medical risk or prior obstetric risk. (DOCX 39 kb)

## Data Availability

No additional data available due to confidentiality restrictions.

## Abbreviations

aOR: Adjusted odds ratio
BC Stats: British Columbia’s Division of Statistics
BC: British Columbia
BMI: Body mass index
GEE: Generalized estimating eq.
GP: General practitioner
HIV: Human immunodeficiency virus
ICD-10-CA: International Statistical Classification of Diseases, Tenth Revision, Canada
LBW: Low birth weight
LHA: Local Health Area
MSP: Medical Services Plan
MW: Midwife/midwifery
OB: Obstetrician
PDR: Perinatal Data Registry
PTB: Preterm birth
SEP: Socioeconomic position
SGA: Small-for-gestational-age birth
USA: United States of America
WHO: World Health Organization

## Footnotes

1 *Indigenous and Northern Affairs Canada* https://www.aadnc-aandc.gc.ca/eng/1100100032463/1100100032464#chp1, and *Statistics Canada* http://www23.statcan.gc.ca/imdb/p3Var.pl?Function=DEC&Id=42932 specify the proper use of the term ‘Status Indian’, as applied in our study.
