# Supplementary material for: Improving birth outcomes for women who are substance using or have mental illness: a Canadian cohort study comparing antenatal midwifery and physician models of care for women of low socioeconomic position
Source: BMC Pregnancy Childbirth. 2019 Aug 6;19:279. doi: 10.1186/s12884-019-2428-y (PMC6683351; doi:10.1186/s12884-019-2428-y)
Supplement: Supplementary file 1 — Sensitivity Analyses. This file (appendix_a_sensitivity_analyses.pdf) contains results of two sensitivity analyses, displayed in Table 1: Adjusted odds ratios and 95% CIs without and with control for antepartum morbidity and Table 2: Adjusted odds ratios and 95% CIs for full study population and for study population excluding pregnancies in which mothers had medical risk or prior obstetric risk. (DOCX 39 kb) [file 12884_2019_2428_MOESM1_ESM.docx]

**Appendix A: Sensitivity Analyses**

**Table 1: Adjusted odds ratios with and without control for antepartum morbidity**

| Antenatal Model | Without Control for Antepartum Morbidity  OR (95% CI) | With Control for Antepartum Morbidity  OR (95% CI) |
| --- | --- | --- |
| Small-for-Gestational-Age Birth (< 10^th^ percentile)^a^ | | |
| MW vs. GP | 0.73 (0.62-0.86) | 0.78 (0.66-0.92) |
| MW vs. OB | 0.60 (0.49-0.72) | 0.68 (0.56-0.82) |
| GP vs. OB | 0.82 (0.73-0.93) | 0.87 (0.77-0.98) |
| Preterm Birth (< 37 weeks gestation)^b^ | | |
| MW vs. GP | 0.79 (0.66-0.94) | 0.86 (0.72-1.03) |
| MW vs. OB | 0.53 (0.43-0.64) | 0.61 (0.50-0.74) |
| GP vs. OB | 0.67 (0.59-0.75) | 0.71 (0.63-0.80) |
| Both models adjusted for maternal age, pre-pregnancy BMI, infant sex, smoking status, substance use, mental illness/disorder, Local Health Area socioeconomic rank  ^a^Model also adjusted for parity  ^b^Model also adjusted for medical risk, prior obstetric risk, receipt of social assistance, neighbourhood SEP, and northern residence | | |

**Table 2: Adjusted odds ratios and 95% CIs for full study population and for study population excluding pregnancies in which mothers had medical risk or prior obstetric risk**

| Antenatal Model | Full study population  OR (95% CI) | Excluding mothers with medical risk or prior obstetric risk  OR (95% CI) |
| --- | --- | --- |
| Small-for-Gestational-Age Birth (< 10^th^ percentile)^a^ | | |
| MW vs. GP | 0.73 (0.62-0.86) | 0.72 (0.61-0.85) |
| MW vs. OB | 0.60 (0.49-0.72) | 0.60 (0.50-0.73) |
| GP vs. OB | 0.82 (0.73-0.93) | 0.84 (0.74-0.95) |
| Preterm Birth (< 37 weeks gestation)^b^ | | |
| MW vs. GP | 0.79 (0.66-0.94) | 0.77 (0.64-0.92) |
| MW vs. OB | 0.53 (0.43-0.64) | 0.53 (0.43-0.65) |
| GP vs. OB | 0.67 (0.59-0.75) | 0.69 (0.60-0.73) |
| Both models adjusted for maternal age, pre-pregnancy BMI, infant sex, smoking status, substance use, mental illness/disorder, Local Health Area socioeconomic rank  ^a^Model also adjusted for parity  ^b^Model also adjusted for medical risk, prior obstetric risk, receipt of social assistance, neighbourhood SEP, and northern residence | | |
